# Biosynthetic Versus Polytetrafluoroethylene Graft in Extra-anatomical Bypass Surgery of Takayasu Arteritis Patients With Supra-aortic Disease

**DOI:** 10.15171/jcvtr.2015.22

**Published:** 2015

**Authors:** Berkan Ozpak, Gokhan Ilhan

**Affiliations:** ^1^ Department of Cardiovascular Surgery, Tekirdag State Hospital, Tekirdag, Turkey; ^2^ Department of Cardiovascular Surgery, Faculty of Medicine, Recep Tayyip Erdogan University, Tekirdag, Turkey

**Keywords:** Takayasu Arteritis, Vascular Surgical Procedures, Tissue Engineered Vascular Graft

## Abstract

*Introduction:* To evaluate treatment outcomes of patients diagnosed with Takayasu arteritis (TA), who underwent extra-anatomical bypass surgery using biosynthetic grafts.

*Methods:* This retrospective study included 12 TA patients considered eligible for surgical revascularization between January 2005 and May 2011 from two vascular surgical units in Turkey. Control group consisted of 12 peripheral arterial disease patients who underwent supra-aortic extra-anatomical bypass surgery using polytetrafluoroethylene (PTFE) graft. Preoperatively, all patients underwent Doppler ultrasound and arteriography. Patients were examined every 3 months for clinical findings after monthly follow-up during the first 6 months, first, second and third year controls. Graft patencies were evaluated by Doppler ultrasound at each visit.

*Results:* The mean age was 38.6 ± 4.2 years and the mean follow-up time was 37.9 ± 6.9 months for the study group. In Biosynthetic Group, subclavian-subclavian (n = 2), axillo-axillary (n =9) and carotico-subclavian (n = 1) bypass operations were performed. In PTFE group, subclavian-subclavian (n = 3), axillo-axillary (n = 7), subclavian-left ulnar (n = 1), subclavian-distal brachial (n = 1) bypass operations were performed. Graft occlusion occurred in four patients in PTFE Group during follow-up period. These occlusive lesions were treated successfully according to the routine of each vascular unit.

*Conclusion:* We concluded that in inflammatory diseases like TA, biosynthetic grafts have promising patency, postoperative clinical findings and lower rates of complications requiring reintervention in mid-term.

## Introduction

Takayasu arteritis (TA) is a rarely seen non-specific chronic vasculitis, which generally involves aorta, and its main branches. Clinical manifestations differ according to the severity of occlusive vascular lesion.^1^ The involved arteries can progress to critical stenosis or occlusion not reversible by medical treatment. The clinical symptoms may differ considerably from one patient to the next; such as decreased or disappeared pulse, bruits, hypertension, renal artery stenosis, aortic regurgitation and pulmonary artery involvement.^2^

TA treatment strategies include immunosuppressive agents or medication with steroids and revascularization procedures.^3^ In TA, the primary treatment is immunosuppression with corticosteroids which results remission in 40%-60% of the patients.^3,4^ Approximately 40% of patients with steroid resistance response to cytotoxic agents^4^ and the percentage of patients who required surgical bypass has been reported to reach 16%-28%.^5^ Biosynthetic grafts, which are more frequently used in recent years, can be an alternative to artery or vein conduits in cases where native arteries are damaged because of inflammation especially in autoimmune diseases.

The aim of this study was to evaluate the mid-term results of biosynthetic grafts, comparing treatment outcomes of 24 patients, who underwent supra-aortic extra-anatomical bypass using biosynthetic and polytetrafluoroethylene (PTFE) grafts.

## Material and Methods

### Study Design

This retrospective multicentre study included TA patients considered indicated for surgical revascularization between January 2005 and May 2011 from 2 vascular surgical units in Turkey. A total of 24 patients were evaluated in 2 groups depending on the graft used: Biosynthetic group (n = 12) and PTFE group (n = 12). As the disease most frequently affects young Asian women of childbearing age there was no significant difference between basic demographic characteristics of two groups.

### Graft Choice

The selection of the graft type was based on the surgeons’ preference at first. However, biosynthetic graft became the first choice of surgeons after availability and excellent short-term results.

**Figure 1:**
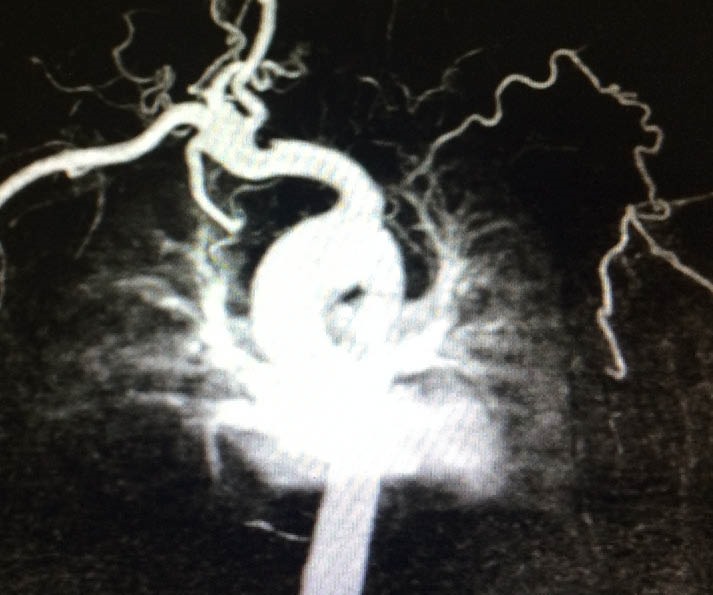
MR angiography image of a 42 year old male TA patient. Both left subclavian and carotid arteries are occluded.

Preoperatively, all patients underwent Doppler ultrasonography and arteriography, with recording of anatomical details of inflow and outflow arteries. Patient characteristics, risk factors, indications for surgery and arteriographic and postoperative findings are shown in Table 1 and Table 2. There were no significant differences between basic demographic characteristics (age, smoking, dyslipidemia, hypertension) of 2 groups. TA was diagnosed according to the American College of Rheumatology Criteria for the Classification of TA.^6^ At least 3 of the following 6 criteria were enough to diagnose our TA patients: Claudication of upper extremities, brachial artery pulselessness or decreased brachial artery pulse, onset age under 40 years, systolic arterial pressure gradient higher than 10 mm Hg between upper extremities, arteriography showing stenosis or occlusion of aorta and its primary branches including large arteries in the proximal upper and lower extremities (Figure 1).

**Table 1:** Clinical Manifestations and Postoperative Findings of Patients Treated With Biosynthetic Grafts

**Age**	**Sex**	**Preoperative Symptoms and Findings**	**MR Angiography Findings**	**Bypass Type**	**Postoperative Symptoms and Findings**
38	F	Left upper extremity claudication	Left subclavian artery stenosis (80%)	Axillo-axillary	Claudication resolved
44	F	Left upper extremity claudication, attacks of cyanosis and pulselessness	Left subclavian artery complete occlusion	Subclavian- subclavian	Claudication resolved
32	F	Left upper extremity claudication	Subclavian + axillary artery stenosis (80%-90%)	Axillo-axillary	Claudication resolved
41	F	Left upper extremity claudication	Left subclavian artery stenosis (90%)	Carotico-subclavian	Claudication resolved
39	F	Left upper extremity claudication, attacks of cyanosis, pulselessness	Left subclavian artery complete occlusion	Axillo-axillary	Claudication resolved, necrosis
39	F	Right upper extremity claudication	Right subclavian artery stenosis (70%-80%)	Axillo-axillary	Claudication resolved,
42	M	Left upper extremity claudication, attacks of cyanosis and pulselessness	Left axillary artery occlusion	Axillo-axillary	Claudication resolved, Cyanotic attacks disappeared
26	M	Right upper extremity claudication	Right subclavian artery stenosis (80%-90%)	Axillo-axillary	Claudication resolved
38	M	Left upper extremity claudication	Left subclavian artery occlusion	Subclavian- subclavian	Claudication resolved
41	M	Cyanosis of the left arm numbness of the left arm + brachiobasilic AV fistula of the right arm	Left subclavian artery proximal occlusion	Axillo-axillary	Claudication and weakness resolved,
35	M	Left upper extremity claudication, attacks of cyanosis, pulselessness	Left subclavian artery short stenotic segment lesion (80%)	Axillo-axillary	claudication resolvedRadial and ulnar pulses recovered.
37	F	Right upper extremity claudication, significant weakness of radial and ulnar pulses	Subclavian artery stenosis (80%-90%)	Axillo-axillary	Numbness of the arm continues. Claudication resolved

Abbreviations: F, female; M, male.

**Table 2:** Clinical Manifestations and Follow-up Findings of Patients Treated With Polytetrafluoroethylene (PTFE) Grafts

**Age**	**Sex**	**Preoperative Symptoms and Findings**	**MR Angiography Findings**	**Bypass Type**	**Postoperative Symptoms and Clinical Findings**
37	F	Left upper extremity claudication	Left subclavian artery stenosis (70%)	Axillo-axillary	Weakness of the arm continues
40	F	Left upper extremity claudication, attacks of cyanosis and pulselessness	Left subclavian artery total occlusion	Axillo-axillary	Claudication resolved.Weakness of the arm continues
36	F	Left upper extremity claudication	Left Subclavian + axillary artery stenosis (80%-90%)	Subclavian- subclavian	Claudication resolved
41	F	Left upper extremity claudication, numbness, significant weakness of radial and ulnar pulses	Left subclavian artery stenosis (90%) Previous aortic valve replacement surgery	Subclavian-subclavian	Graft occlusion occurred at sixth month
44	F	Right upper extremity claudication, significant weakness of radial and ulnar pulse	Right subclavian artery stenosis (70%)	Subclavian- subclavian	Graft occlusion occurred at 24th month
47	F	Left upper extremity claudication	Left subclavian artery stenosis (80%-90%)	Axillo-axillary	50 mm Hg pressure difference
42	F	Right upper extremity claudication, significant weakness of radial and ulnar pulse	Right subclavian artery stenosis (70%)	Axillo-axillary	Graft occlusion occurred at 31st
39	F	Left upper extremity claudication,	Left subclavian artery short stenotic segment lesion(80%)	Axillo-axillary	Weakness of brachial pulse continues.
38	F	Left upper extremity claudication	Left subclavian artery stenosis (70%)	Axillo-axillary	Graft occlusion occurred at 19th month
34	F	Left upper extremity claudication	Left axillary artery stenosis	Axillo-axillary	Graft occlusion occurred at 23rd month
39	M	Right upper extremity claudication, significant weakness of radial and ulnar pulse	Right subclavian artery stenosis (70%-80%)	Left subclavian – left ulnar	Claudication resolved. Left ulnar pulse present, radial pulse absent.
38	M	Left upper extremity claudication, no radial and weak ulnar pulse	Total occlusion of left subclavian artery	Right subclavian –right distal brachial	Claudication resolved. Right radial and ulnar pulse present

Abbreviations: F, female; M, male.

Surgery was performed using end-to-side anastomosis in all cases both proximally and distally.

The patients who failed medical therapy were considered for operation. This included the patients with acute occlusive or claudication symptoms and chronic symptoms with under maximum medical therapy. All revascularizations were performed by open surgical technique using biosynthetic or PTFE graft. No balloon or stenting was applied. Non-inflamed arterial districts were chosen for both anastomotic sites. Regional inflow and outflow of bypass anastomotic sites were evaluated peropertively to maintain maximum graft patency. Perioperative antithrombotic therapy and antibiotics were administered according to the routine of the individual vascular unit. All patients were administered 100 mg acetylsalicylic acid at the first postoperative day of operation.

The biosynthetic graft used in this study; Omniflow II (Bio Nova International Pty Ltd, North Melbourne, VIC, Australia) is a collagen-polyester composite that has been successfully used for peripheral vascular replacement.^7^

### Outcome Parameters

Patients were examined for clinical findings monthly during the first 6 months. All patients were invited for the follow up visit 10 days after the discharge, and their outcomes and complaints were recorded. Graft patency was evaluated with arterial ultrasonography at sixth month, first, secondand third year controls.

## Results

A total of 24 patients met the eligibility criteria for the study. Of the 24 patients (17 females, seven males), the mean age was 38.6 ± 4.2 (range 26 to 47) years. The mean duration of follow-up for the study group was 37.9 ± 6.9 months. Both biosynthetic and PTFE groups included 12 patients. The mean age was 37.6 ± 4.81 (range 26 to 44) years for Biosynthetic group, and 39.5 ± 3.5 (range 34 to 47) years for PTFE group. The mean follow-up time was 37.6 ± 4.8 months for Biosynthetic group, and 39.5 ± 3.5 months for PTFE group.

The patients presented with various complaints as impaired vision, fainting, weakness of arm muscles, and numbness. Physical examination revealed hypertension, and differences in blood pressures, and pulse rates between both extremities.

In Biosynthetic group, subclavian-subclavian (n = 2), axillo-axillary (n = 9) and carotico-subclavian (n=1) bypass operations were performed. In PTFE group, subclavian-subclavian (n = 3), axillo-axillary (n = 7), subclavian-left ulnar (n = 1), subclavian-distal brachial (n = 1) bypass operations were performed. In PTFE group, 4 patients developed recurrent claudication. Three patients who underwent subclavian-subclavian bypass surgery developed graft occlusion at 6th, 23th and 24th months and 2 patients who had axillo-axillary bypass surgery developed graft occlusion at 19th and 31st months. These occlusive lesions in 5 patients were treated according to the routine of each vascular unit . Vascular revascularization was successfully achieved in all cases. No early or late infection, major amputation, or perioperative death was observed in both groups.

## Discussion

TA is a chronic large vessel vasculitis of unknown etiology. The histopathological appearance, involving all the arterial walls, is characterized by granulomatous inflammation and “panarteritis” with developed intimal fibrous thickness.^8^ This inflammation leads to arterial stenosis, thrombosis and aneurysm. Prognosis changes depending upon the vascular involvement and arterial inflammation. Extensive stenosis/occlusion depending on the involvement of extremity arteries and associated claudication were present in our cases. Upper extremity pulses in involved area were poor or not palpated, and upper extremity ischemia symptoms like cyanosis, numbness including claudication were dominant symptom, dominant symptoms. Arteriography is considered as the gold standard in clinical diagnosis and classification of TA.^9^ Contrast-enhanced computed tomography angiography and particularly magnetic resonance angiography can demonstrate arterial anatomy, wall enhancement, edema, and thickening which might enable early disease detection where luminal diameter is still preserved.^10^ We preferred to use both magnetic resonance angiography and digital subtraction angiography to monitor all TA patients and observed a common involvement of the upper and lower extremity.

To avoid complications, the surgical or endovascular procedure is recommended when the disease is dormant or inactive.^1^ In a comprehensive study conducted by Saadoun et al, surgical revascularization conducted when the inflammation markers are at their highest level have more complications compared to multivariate analysis results.^11^ Some have claimed that surgical or endovascular interventions should be avoided during the acute inflammatory stage of TA to avoid anastomotic dehiscence or restenosis. According to Fields et al,^12^ performing surgery in patients with active-stage TA increases the risk of early graft revision and the progression of symptomatic disease in other arterial beds. In this study to assess TA disease activity, C-reactive protein (CRP) level was used as biologic marker of inflammation along magnetic resonance imaging (MRI). Active-stage TA was defined as one or more of the following: CRP (>0.9 mg/dL), arterial wall thickening on MRI or the presence of systemic symptoms. We used C-reactive protein levels for both treatment, timing of surgery. It appears that patients with CRP levels below 1 mg/dL had an excellent long-term outcome, but 4 patients with 1mg or higher had undergone surgery because of emergency circumstances and close long-term follow-up was required in these patients. However, in severely symptomatic TA patients with SAA lesions, it is not always possible to postpone SAA reconstruction until biologic markers return to normal. On the other hand, these four patients had biosynthetic graft and when assesed by MR angiography grafts’ anastomotic sites were not affected and the disease in the extremities did not progress. Postoperative anti-inflammatory therapy was performed for these patients.

Pharmacologic therapy is the primary therapy for TA, but surgical or endovascular treatment may be required to treat organ ischemia, renovascular hypertension, or aneurysmal lesions in late-stage TA.^12^ However, an evidence-based consensus regarding indications for surgical or endovascular intervention and optimal treatment options for patients with TA involving SAA lesions are lacking.

The multiplicity of progressive lesions is challenging in the management of TA, with endovascular treatment taking priority over surgery. In TA, unlike atherosclerotic lesions, the vessels are firm and fibrotic. Thus, arch vessels require higher balloon inflation pressure. However, this challenging anatomical structure of the lesion in TA becomes the main reason for technical failure in subclavian artery stenosis. On the other hand, although endovascular treatment can be attempted as a less-invasive treatment for short stenotic subclavian artery lesions this treatment is associated with a higher risk of restenosis or occlusion than bypass surgery, even after postoperative anti-inflammatory treatment.^13^ In another recent study the results for endovascular intervention (angioplasty and stenting) are less encouraging in comparison and surgical treatment had a better outcome compared to endovascular intervention in terms of postoperative complications (37.5% and 50%, respectively).^11^ Long-term survival rates following surgical bypass procedures are good in recent studies.^12,14^ Cong et al^5^ echo this, where restenosis occurred in 34.7% of surgical bypasses and 77.3% of angioplasty procedures. In the light of previous studies it is quite difficult to compare percutaneous transluminal angioplasty (PTA) and extra-anatomic bypass procedures to each other since the subclavian artery stenosis requiring treatment is seldom observed. Aburahma et al^15^ compared 51 patients who had carotico-subclavian bypass (CSB) to 12 patients who had PTA in 2007. One, 3 and 5 years patency was 100%, 98% and 96% for the CSB group and 93%, 78% and 70% for PTA group (*P *< .0001).On the other hand, Sharma et al^16^ reported a technical success of 89% in 66 TA patients who had undergone endovascular treatment. Restenosis rate was 16% at 22 months.^16^ Also a cohort study conducted recently resulted in disappointment from an endovascular procedures point of view. Patients who had both endovascular and surgical revascularizations had good initial results, while 78% of patients on whom endovascular revascularization is applied and 36% of patients who underwent surgical revascularization developed restenosis.^17^ In another study by Liang et al^18^ a high failure rate for endovascular revascularization procedures to treat various arterial lesions in TA patients was reported.Surgical reconstruction is mainly performed to improve brain ischemic symptoms or to prevent stroke in patients with SAAs caused by TA.^19^ Generally, indications for surgery of TA include hypertension with critical renal artery stenosis, extremity claudication limiting activities of daily living, cerebrovascular ischemia or critical stenosis of three or more cerebral vessels, moderate aortic regurgitation, and cardiac ischemia with confirmed coronary artery involvement.^3^ Extremity claudication was the most common symptom found in our patients. In the present study arterial occlusion was most common in the left subclavian artery.

In anatomical locations where bypass grafting is not possible or hazardous, extraanatomical bypass methods can be preferred to ensure blood supply to the ischemic region.^20^ TA patients included in this study have been operated using various extraanatomical bypass procedures in consideration of the location of the lesion and general health state of the patients.

For upper extremities, extra-anatomic bypass methods are utilized for revascularization since innominate and subclavian arteries are restricting, and bypass operations conducted through an anatomic path within thorax are associated with a high mortality and morbidity rate.^21,22^ Possible paths of extra anatomical bypass graft are CSB, carotid-subclavian transposition, axillo-axillary bypass and subclavian-subclavian bypass. Not many detailed studies regarding these procedures are present and in the existing studies primary and secondary patency rates are varying between 82% to 100%.^23-25^ Kieffer et al^26^ reported satisfactory early and long-term patency of 24 TA patients who were operated due to renal artery stenosis. In the follow-up period averaging 61.3 months, only 4 patients required secondary revascularization procedures.

**Figure 2:**
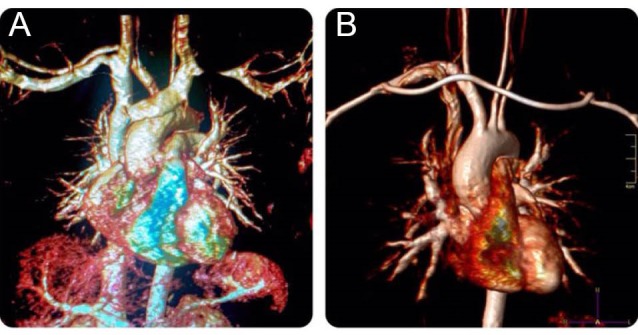
Preoperative and postoperative(axillo-axillary bypass) MR angiography image of a 39-year-old female.

**Figure 3:**
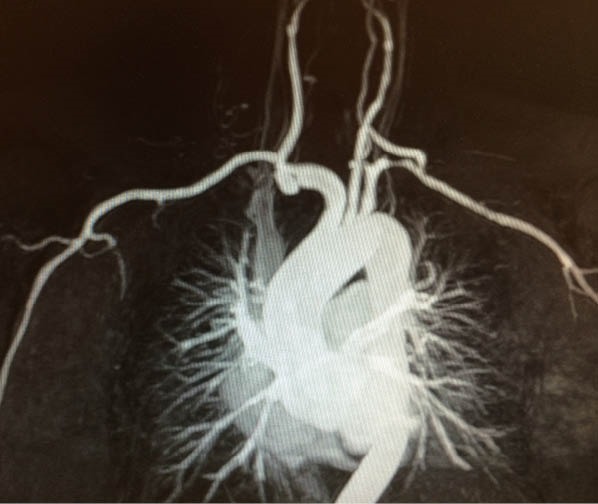
Postoperative (carotico-subclavian bypass) MR angiography image of a 41-year-old female patient.

Axillo-axillary bypass, one of our techniques of choice (Figure 2A, B), is an advantageous surgical technique, because it is simple and supraclavicular incision and temporary carotid occlusion are not needed. In a previous study, in which long-term follow-up of axillo-axillary bypass applied in 32 cases, 3- and 10-year graft patency rates have been reported as 95% and 73%, respectively.^27^ Thus, we used axillo-axillary bypass in nine of 12 TA patients due to the compatibility of lesion areas. Comparative studies between CSB and axillo-axillary bypass revealed contradictory data about long-term patency rates. However, mortality and morbidity rates of both operations are close to each other.^28^ Linni et al^29^ compared PTA and CSB and reported that vessels with PTA were occluded in 48% of the patients with subclavian artery occlusion, while all CSB grafts maintained their patency. We preferred CSB procedure in one TA patient with a brachiobasilic fistula in one arm without contralateral carotid stenosis and achieved total graft patency for one year (Figure 3).

Graft material chosen in graft interpositioning is an important factor that affects patency rate. The biosynthetic vascular graft used in this study (Omniflow II^®^) is composed of bovine collagen stabilized with glutaraldehyde, and has high biocompatibility due to biosynthetic material in its content. Polyester mesh also provides graft wall microvascularization.^23^ On the other hand; biosynthetic material is also an attractive long-term option due to its bio-compatibility allowing the integration of the graft into host tissues as well as true micro-vascularisation of the graft vessel wall. Immunohistological studies of biosynthetic grafts show that original bovine collagen is still present in the graft and it is strengthened with the host-related connective tissue even after 4 years following the transplantation of graft.^30^

Surgical outcomes have improved over time, aided by advances in surgical techniques as well as improvements in graft technology. Our study about the use of biosynthetic grafts for TA patients has some limitations. Although our study is limited with small number of subjects, we assessed the short and midterm results of biosynthetic graft for supra-aortic arterial lesions in patients with TA. However, it was reassuring to achieve complete patency in biosynthetic graft group. A recent **s**tudy by Ziomek et al^31^ documented that prosthetic grafts had a better patency than saphenous vein grafts at thia arterial district. Furthermore, it was reported that ringed PTFE grafts were more durable than Dacron grafts by previous studies.^32,33^ However, we could not find a study comparing biosyhnthetic and PTFE graft at this location.

In conclusion, we assessed encouraging results in management of arterial lesions with biosyhnthetic grafts at supra-aortic location of this special patient group progressing with inflammation attacks, in that they have satisfactory short and midterm graft patencies, and lower rates of complications (ie, restenosis) requiring re-intervention.

## Ethical issues

None to be declared.

## Competing Interests

Authors declare no conflict of interest in this study.
